# Branched-Chain Amino Acid Catabolism Promotes Ovarian Cancer Cell Proliferation via Phosphorylation of mTOR

**DOI:** 10.1158/2767-9764.CRC-24-0532

**Published:** 2025-04-07

**Authors:** Hannah J. Lusk, Monica A. Haughan, Tova M. Bergsten, Joanna E. Burdette, Laura M. Sanchez

**Affiliations:** 1 Department of Chemistry and Biochemistry, University of California Santa Cruz, Santa Cruz, California.; 2 Department of Pharmaceutical Sciences, College of Pharmacy, University of Illinois Chicago, Chicago, Illinois.

## Abstract

Ovarian cancer is the sixth leading cause of cancer-related mortality among individuals with ovaries, and high-grade serous ovarian cancer (HGSOC) is the most common and lethal subtype. Characterized by a distinct and aggressive metastatic pattern, HGSOC can originate in the fallopian tube with the transformation of fallopian tube epithelial (FTE) cells, which metastasize to the ovary and subsequently to the omentum and peritoneal cavity. The omentum is a privileged metastatic site, and the metabolic exchange underlying omental metastasis could provide enzyme or receptor targets to block spread. In this study, we adapted a mass spectrometry imaging (MSI) protocol to investigate spatial location of three-dimensional cocultures of tumorigenic FTE cells when grown in proximity to murine omental explants as a model of early metastatic colonization. Our analysis revealed several altered metabolites in tumorigenic FTE/omentum cocultures, namely changes in branched-chain amino acids (BCAA), including valine. We quantified the heightened consumption of valine, other BCAAs, and other amino acid–derived metabolites in omental cocultures using LC/MS assays. Our analysis revealed that metabolite concentrations when monitored with MSI from cell culture media in living culture systems have notable considerations for production of signatures by MSI data that induce ionization suppression. Supplementation with valine enhanced proliferation and mTOR signaling in tumorigenic FTE cells, suggesting the potential of BCAAs as a nutrient utilized by tumor cells during omental colonization and a possible target for metastasis.

**Significance:**

This study uncovers altered amino acid metabolism, specifically increased BCAA catabolism, at the interface of ovarian cancer cells and omental tissue in a coculture model of HGSOC secondary metastasis. Enhanced BCAA catabolism promotes cancer cell proliferation through mTOR signaling, presenting potential therapeutic value. These findings deepen our understanding of HGSOC pathogenesis and the metastatic tumor microenvironment, offering insights for developing new treatment strategies.

## Introduction

Ovarian cancer is the most lethal gynecologic malignancy (1). Projections indicate that in 2024, the United States will see 19,680 new cases and 12,740 deaths attributed to ovarian cancer (1). Despite the term “ovarian cancer” implying a singular condition, it encompasses several histologically distinct subtypes; among them, high-grade serous ovarian cancer (HGSOC) is the most prevalent and lethal (2). HGSOC poses challenges in diagnosis due to nonspecific symptoms and the absence of efficient screening methods (2–4). Because of a lack of routine screening, approximately 80% of cases remain undetected until reaching metastatic stages when the 5-year survival rate drops below 30% (2, 3, 5). These data underscore the need to investigate the molecular events driving HGSOC pathogenesis to facilitate the discovery of untapped therapeutic targets.

The metastatic behavior of HGSOC suggests that the omentum is a unique metastatic location, with omental metastases observed in approximately 80% of cases (6, 7). Several studies provide evidence for an “activated” phenotype of the peritoneal microenvironment associated with ovarian cancer, suggesting that chemical messengers specifically account for the preferential spread of fallopian tube–derived HGSOC to the omental tissues during metastasis. The omentum is comprised of a variety of cell types, including adipocytes, fibroblasts, and macrophages, which can be dynamically converted to “cancer-associated” phenotypes, which is a process known to play a role in disease progression (8–10). For example, following coculture with ovarian cancer cells, activated omental adipocytes have been shown to enhance the secretion of various adipokines, thus facilitating ovarian cancer dissemination (7). A study by Rizi and colleagues demonstrated that cancer-associated adipocytes can also release arginine, which ovarian cancer cells may uptake, leading to increased nitric oxide production. Consequently, ovarian cancer cells release citrulline, a byproduct of nitric oxide synthase activity, which promotes adipogenesis in adipocytes (11). Furthermore, cancer-associated adipocytes have been reported to transfer fatty acids to ovarian cancer cells, thereby promoting β-oxidation and energy generation which ultimately drive metastatic growth (7). Ovarian cancer cells also secrete soluble metabolites and microRNA that induce metabolic reprogramming in fibroblasts, prompting the acquisition of cancer-associated phenotypes. For instance, a study by Jeon and colleagues (12) unveiled that lysophosphatidic acid, found in the malignant ascites of patients with advanced-stage ovarian cancer, triggers the differentiation of adipose-derived mesenchymal stem cells into cancer-associated fibroblasts. Bidirectional interactions between ovarian cancer cells and other cell types in the microenvironment such as fibroblasts and adipocytes have been reported in several studies (13–16). Furthermore, the changes conferred by fibroblasts and adipocytes have been shown to promote metastasis and chemoresistance in ovarian cancer (16, 17). Inspired by these findings, we seek to further understand the chemical signaling in ovarian cancer.

Although we have some knowledge about the role secreted local factors play in omental colonization (14, 18), the role of secreted metabolites, their spatial resolution, and their quantification have typically not been studied utilizing an untargeted label-free approach to detect the chemical cues driving this critical step in metastasis. Mass spectrometry imaging (MSI) is a useful tool for visualizing the spatial distribution of metabolites in biological samples (19, 20). Our lab previously developed an MSI protocol for analyzing three-dimensional (3D) cocultures of explant murine tissues and mammalian cells and discovered and validated the presence of norepinephrine as a chemoattractant released from the ovary (21, 22). Furthermore, our labs demonstrated that norepinephrine release was partially mediated by SPARC and affected survival in a murine model (23, 24). In this present study, we adapted our MSI technique for use with omental tissue to investigate metabolic exchange in secondary metastasis.

MSI analysis of 3D omental cocultures led to the identification of several signals that seemed to be altered in tumorigenic conditions. MSI on cocultures in a divided chamber format revealed the site of origin of these signals, with some signals originating from tumorigenic fallopian tube epithelial (FTE) cells and others originating from omental tissue. Further analytic validation of *m/z* 118 revealed that it represented the branched-chain amino acid (BCAA) valine. Quantification of BCAAs and 21 other amino acids in coculture extracts demonstrated that valine and other BCAAs are actually consumed at higher levels in tumorigenic FTE/omentum cocultures. Analysis of valine standard curves via MSI illustrated that the observed increase in signal was attributed to ion suppression, owing to the high concentration of valine in the media. Proliferation assays revealed that valine supplementation increases the proliferation of tumorigenic FTE cells, and immunoblotting showed that valine can stimulate the phosphorylation of mTOR. This work demonstrates the benefit of exploring spatial metabolic changes in cancer metastasis.

## Materials and Methods

### Mouse colony and omentum removal

All animals were treated in accordance with NIH Guidelines for the Care and Use of Laboratory Animals and the established Institutional Animal Use and Care protocol #22-188 at the University of Illinois Chicago. Omental tissue was collected from female CD-1 mice 6 to 8 weeks old (Charles River Laboratories). Mice were housed in a temperature- and light-controlled environment (12 hours light and 12 hours dark) and provided food and water *ad libitum*. Omentums were removed immediately after sacrifice and bisected to produce tissue pieces of consistent size using a dissecting microscope (Leica MZ6).

### Cell lines

Dr. Barbara Vanderhyden from the University of Ottawa generously provided spontaneously immortalized murine ovarian surface epithelial (MOSE) cells and murine oviductal epithelial cells (MOE), which are equivalent to human FTE. These MOE cells were modified in our laboratory to express different genetic variations, including a scrambled control (SCR) short hairpin RNA (shRNA; MOE SCR^shRNA^) and an shRNA targeting the *PTEN* gene (MOE PTEN^shRNA^), in which case the reduction of PTEN induces tumorigenic phenotype (25). Cells were passaged a maximum of 30 times. Cell lines were authenticated via short tandem repeat validation by DNA Diagnostics Center. Engineered modifications were confirmed and were regularly assessed upon thawing and greater than 15 passages by Western blotting. *Mycoplasma* testing was done using Sigma LookOut Mycoplasma PCR Detection Kit and Lonza MycoAlert Mycoplasma Detection Kit.

MOSE cells were cultured in minimum essential medium α (αMEM; MT10022CV, Thermo Fisher Scientific) supplemented with 10% FBS (21G267, Sigma-Aldrich), 2 mmol/L L-glutamine (TCG0063, VWR), 10 mg/mL insulin-transferrin-sodium selenite supplement (11074547001, Sigma-Aldrich), 1.8 ng/mL EGF (100-15, PeproTech), 100 U/mL penicillin–streptomycin (15140-122, Gibco), and 1 mg/mL gentamicin (30-005-CR, CellGro). The MOE cell lines (SCR^shRNA^ and PTEN^shRNA^) were maintained in similar media but with the addition of 18.2 ng/mL estradiol-17β (E1024, Sigma-Aldrich) and selection antibiotics.

### Coculture incubation for downstream MSI analyses

Low-melting agarose (2%, A9414, Sigma-Aldrich) was liquified from a solid at 70°C. Cells maintained in αMEM in T-75 flasks were rinsed with PBS (16777, VWR) and detached using 1× trypsin (25200072, Life Technologies). The detached cells were transferred to 10 mL αMEM and counted using an automated cell counter (Accuris E7500 QuadCount). Aliquots of the cells in αMEM were transferred to centrifuge tubes and centrifuged for 5 minutes at 900 rpm (Eppendorf 5804 R Benchtop Centrifuge). After removing the αMEM, cell pellets were resuspended in 2× DMEM (D5523, Sigma-Aldrich) supplemented with 10% FBS and 2× penicillin–streptomycin to a concentration of 333 cells/μL. These cell suspensions were mixed 1:1 with 2% agarose to yield a final cell concentration of 166 cells/μL in 1% agarose and 1× DMEM.

Halved omental explants were placed in the corner of wells in an eight-well chamber (177445, Lab-Tek) attached to an indium tin oxide-coated glass slide (8237001, Bruker Daltonics). Each condition (300 μL) was plated into a well, ensuring the tissue remained in the corner of the well. For the divided chamber layout, a removable plastic divider was placed diagonally across each well (21, 22). Cells in agarose were plated first (150 μL) on one side of the divider. After the agarose solidified, the divider was removed, and tissue in agarose (150 μL) was plated on the other side.

The slides were placed in a humidified incubator at 37°C and 5% CO_2_ and cocultured for 4 days. After 4 days, the eight-well chamber was detached from the glass slide, and omental tissue was removed with a razor blade prior to desiccation. Agarose cocultures were desiccated on the indium tin oxide-glass slide in an incubator at 30°C on a home-built spinning apparatus for 4 hours (26).

### Matrix application

Matrix-assisted laser desorption ionization (MALDI) matrices α-cyano-4-hydroxycinnamic acid [CHCA (98%), C2020, Sigma-Aldrich] and 2,5-dihydroxybenzoic acid [(98%), 149357, Sigma-Aldrich] were recrystallized in-house as previously described (22). The MALDI matrix used for MSI was a 50:50 mixture of CHCA:2,5-dihydroxybenzoic acid at 10 mg/mL dissolved in 90:10 acetonitrile (ACN):H_2_O with 0.1% trifluoroacetic acid applied using a TM sprayer (HTX Imaging).

### MSI analysis

Before MSI analysis, slides were scanned using Tissue Scout (Bruker Daltonics) for the initial screen and Epson Perfection V850 Pro for subsequent experiments. Scanned images were used to guide irradiation. MSI data were acquired using timsControl v2.0.51.0_9669_1571 and flexImaging 5.1 software for the initial screen and flexImaging 7.4 for subsequent experiments at 100 μm spatial resolution on a timsTOF fleX mass spectrometer (Bruker Daltonics). Data were collected using a mass range of 50 to 1,500 Da in positive ion mode with the laser width set to 100 μm imaging and the laser power set to 90%. At each raster point, 1,000 laser shots were delivered at a frequency of 1,000 Hz. The instrument was calibrated manually using phosphorus red prior to imaging. MSI data were analyzed, and statistical analysis was performed using SCiLS Lab version 2023c core (Bruker Daltonics). All spectra were normalized to the root mean square. These data are available at MassIVE under accession number MSV000095459.

### Extraction of 3D cocultures for signal validation and quantification

Cocultured agarose plugs were dried *in vacuo* and macerated with a sterile toothpick prior to extraction with 4 mL 50:50 dimethylformamide:H_2_O with 0.1% formic acid (FA). Samples were sonicated for 1 hour before the extract was filtered through a 0.2-μm nylon filter (09719C, Thermo Fisher Scientific), and the supernatant was dried *in vacuo*.

### MALDI/tandem mass spectrometry of coculture extracts

Coculture extracts were normalized by dry weight and resuspended in 50:50 MeOH:H_2_O at 1 mg/mL. A valine analytic standard (BP397, Fisher Biotech) was prepared at a concentration of 0.1 mg/mL in 50:50 MeOH:H_2_O. Extracts and analytic standards were mixed 1:1 with the CHCA matrix (40 mg/mL in 78:22 ACN:H_2_O with 0.1% trifluoroacetic acid) and spotted on an MTP target plate (8280784, Bruker Daltonics). MALDI/tandem mass spectrometry data were acquired using timsControl v2.0.51.0_9669_1571 on a timsTOF fleX mass spectrometer (Bruker Daltonics). Data were collected using a mass range of 50 to 650 Da in positive ion mode with the laser width set to 100 μm using an M5 defocus laser. The laser power was set to 60%. At each raster point, 200 laser shots were delivered at a frequency of 1,000 Hz. The collision energy used for fragmentation was 20 eV. The instrument was calibrated manually using phosphorus red prior to analysis. Data were analyzed using DataAnalysis version 6.1 (Bruker Daltonics).

### LC/MS analysis using aTRAQ kit to quantify amino acids

Coculture extracts were resuspended in H_2_O at 1 mg/mL, and amino acids were quantified using an aTRAQ kit for amino acid analysis of physiologic fluids (Sciex). Coculture extracts (10 μL) were derivatized using the protocol and reagents specified by the kit, dried *in vacuo*, and resuspended in 30 μL H_2_O (27, 28). Reverse-phase liquid chromatography was performed on an Elute UPLC (Bruker Daltonics) using an amino acid analyzer C18 column (5 μm, 4.6 mm × 150 mm, 4374841, Sciex) with a sample injection volume of 2 μL. The mobile phase consisted of A (H_2_O with 0.1% FA and 0.01% hepatofluorobutyric acid) and B (ACN with 0.1% FA and 0.01% hepatofluorobutyric acid) with a flow rate of 0.4 mL/minute. The gradient began with 2% B and was linearly increased to 40% B over 12 minutes; 40% B was held for 8 minutes and then 40% B was linearly increased to 90% B over 2 minutes. The column was washed with 90% B for 2 minutes before linearly decreasing to 2% B over 2 minutes. The column was re-equilibrated with 2% B for 10 minutes. The temperature of the column oven was 50°C. Mass spectrometry spectra were collected using a timsTOF fleX mass spectrometer (Bruker Daltonics) in positive ion mode with a mass range of 50 to 850 Da and a spectra rate of 4 Hz. Prior to analysis, the instrument was calibrated using 0.5 mmol/L sodium formate. All samples were analyzed in three biological replicates (*N* = 3). A normalized response was calculated using the ratio of the AUC of the analyte peak to the internal standard peak. The concentration of amino acids in coculture extracts was determined by multiplying this normalized response by the concentration of the corresponding internal standard for each amino acid. As samples were diluted 3× prior to analysis, this result was multiplied by three to determine concentrations in the original 1 mg/mL extract. These data are available at MassIVE under accession number MSV000095455.

### Proliferation assay

Cells were plated at a density of 1,000 cells per 100 μL in 96-well plates. The cells were allowed to attach to the plate for 24 hours prior to treatment. Plates were fixed with 20% trichloroacetic acid on days 0 and 3 or 5 depending on the cell lines’ growth rate. MOE PTEN^shRNA^, MOE SCR^shRNA^, and MOSE cells were collected after 5 days whereas MOE PTEN^shRNA^ p53^R273H^ was collected after 72 hours. Cell viability was then determined using 0.04% sulforhodamine B via colorimetric detection at 505 nm. (29) All data were normalized to day 0.

### Immunoblotting

A total of 250,000 cells were seeded into six-well plates and left to grow for 24 hours prior to treatment. The cells were removed via trypsin–EDTA, spun down, and resuspended for lysis in RIPA buffer (50 mmol/L Tris pH 7.6, 150 mmol/L NaCl, 1% Triton X-100, and 0.1% SDS) with protease (04693159001, Roche) and phosphatase inhibitors (524625, MilliporeSigma). Protein concentration was determined by Bradford assay (5000006, Bio-Rad). The protein lysate (30 μg) was loaded onto an SDS-PAGE gel and transferred to a nitrocellulose membrane. Blots were blocked with 5% BSA in tris buffered saline with Tween-20 and probed at 4°C overnight with primary antibodies, washed thrice, incubated with secondary antibody for 30 minutes, washed thrice, and then developed as described previously (30). Primary antibodies were utilized at a concentration of 1:1,000 and included the following: mTOR (CST 2983), p-mTOR (CST 2971), and GAPDH (CST 2118). Secondary antibody was anti-rabbit and horseradish peroxidase linked (7074S, Cell Signaling Technology) and used at a concentration of 1:10,000. The blots were analyzed using ImageJ. Results were normalized first to the loading control followed by normalization to the total mTOR protein for phosphorylated mTOR.

### Statistical analysis

Ion images were analyzed using SCiLS Lab image analysis software (Bruker Daltonics) using the “Find values colocalized to regions” feature. Each experimental condition was set as a different region of interest, and the software was used to find signals colocalized to the MOE PTEN^shRNA^ + omentum region compared with all other regions. The software uses Pearson correlation analysis and considers only statistically significant correlations, in which statistical significance (*P*) is defined as *P* = 0.05. This method for the analysis of MSI images is described in McDonnell and colleagues (31) LC/MS quantification and proliferation data were analyzed by one-way ANOVA with Tukey *post hoc* with a cutoff of 0.05 for significance. All statistically significant comparisons are marked (*, *P* < 0.05). Densitometry data were analyzed by a Student *t* test (*, *P* < 0.05). Error bars presented for quantification of LC/MS data, proliferation, and densitometry represent SEM.

### Data availability

The MSI raw data and imzML files from study are publicly available in MassIVE under accession number MSV000095459. The LC/MS data generated in this study are publicly available in MassIVE under accession number MSV000095455. Other data generated in this study are available within the article and its supplementary data files.

## Results

### MSI coculture adaptation for use with omental tissue

**Figure 1:**
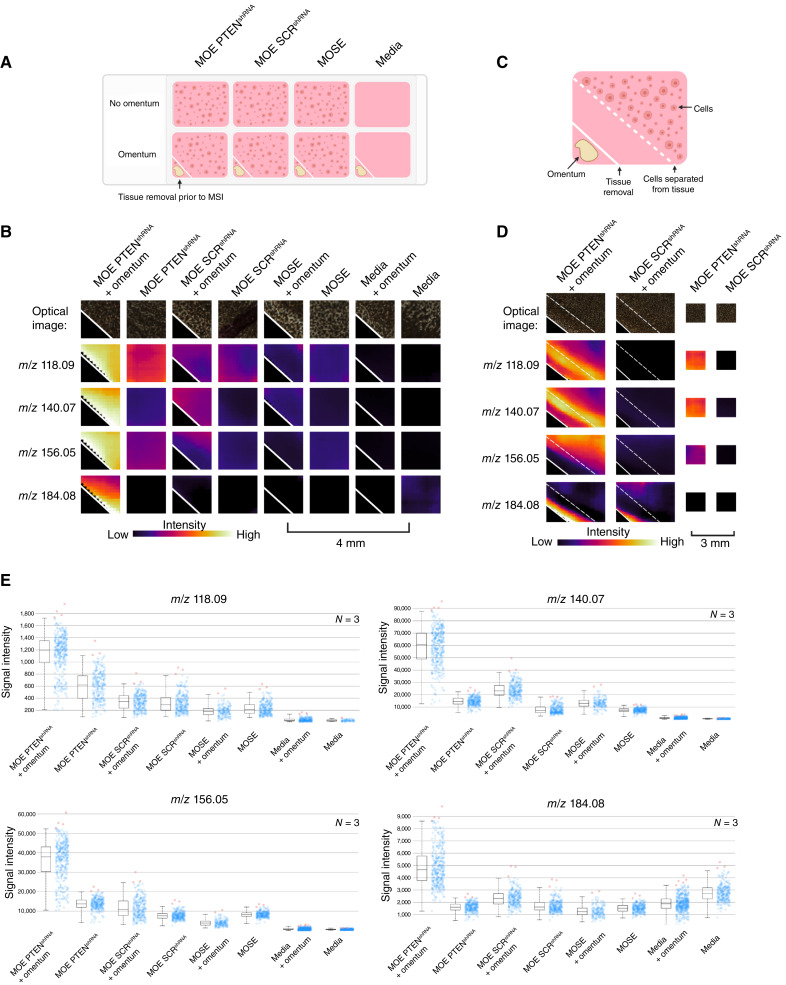
MSI analysis shows several signals specific to the tumorigenic FTE cell/omentum coculture condition. **A,** Illustration of imaging layout on the slide for untargeted analysis, including tumorigenic FTE cells (MOE PTEN^shRNA^) and nontumorigenic controls. **B,** Ion images showing signal intensity of four representative signals upregulated in the HGSOC condition specific to the PTEN mutation (*N* = 3). **C,** Illustration of the divided well layout to identify the origin of signals. **D,** Signals in **B** are replicated when cells and omentum are physically separated in agarose. Signals at *m/z* 118, *m/z* 140, and *m/z* 156 originate from tumorigenic FTE cells, whereas the signal at *m/z* 184 originates from omental tissue. **E,** Boxplots of MSI signals *m/z* 118, *m/z* 140, *m/z* 156, and *m/z* 184. Red and blue dots represent individual pixels, and red dots are outliers. These signals are statistically significant compared with MOE PTEN^shRNA^ + omentum using Pearson correlation analysis (*P* < 0.05). [**A** and **C,** Created in BioRender. Sanchez, L. (2025) https://BioRender.com/cy6a9xj.]

Although our MSI method was originally envisioned as a plug-and-play technique that could be adapted to many biological cell cultures and organoid cultures, adaptation of this protocol for use with omental tissue was necessary. MALDI-MSI requires a flat sample (32, 33), and because of its fatty nature, omental tissue does not dry flat but crystalizes in heat when desiccated (Supplementry Fig. S1). To address this inconsistent omental crystallization, we placed the tissue in the corner of the eight-well chamber, rather than the center as we have previously reported with murine ovary explants, and removed the omental tissue from cocultured agarose plugs prior to sample desiccation and MSI analysis (Fig. 1A). We confirmed that the omental tissue is viable after 4 days of incubation in agarose (Supplementry Fig. S2).

### Initial MSI screen revealed several signals with increased ionization in tumorigenic FTE cell/omentum coculture and the origin of these signals

We chose to utilize an MOE cell line with an shRNA targeting the *PTEN* gene (MOE PTEN^shRNA^) as our tumorigenic FTE cell model because we have previously demonstrated that silencing PTEN was sufficient to drive colonization of both the ovary and the omentum *in vivo* using athymic mice (34). This model is advantageous because the omental tissue and FTE cells are from the same species, eliminating species differences. In the experimental design for the initial screen, we included several controls, including an MOE cell line expressing a scrambled control shRNA (MOE SCR^shRNA^) to test for cells that are not tumorigenic, an MOSE cell line to test for cell specificity, and a media-negative control. Each condition was cultured alone and cocultured with omental tissue (Fig. 1A). The images are pseudo-colored by the intensity of the ion with spatial referencing, demonstrating the intensity of the signal between the cells and omentum. Samples were incubated for 4 days to allow interaction to occur prior to imaging (*N* = 3). Samples were analyzed using a MALDI quadrupole time-of-flight mass spectrometer.

The original MSI screen yielded more than 25 signals specific to the tumorigenic FTE cell/omentum coculture condition, of which four representative signals are depicted in Fig. 1B and E and replicated in Supplementry Figs. S3 and S4. Some sample heterogeneity was observed among replicates, likely because omental tissues were extracted from different mice for each experiment. These signals were determined to have significantly increased ionization (*P* < 0.05) using the MSI data analysis software SCiLS when comparing MOE PTEN^shRNA^ + omentum against all other conditions, including scrambled + omentum (35). Additional MSI using a divided chamber coculture format (Fig. 1C) revealed the origin of signals and confirmed signals that accumulate in agarose without direct physical contact between the cells and the tissue. Three signals (*m/z* 118, *m/z* 140, and *m/z* 156) were found to be produced by tumorigenic FTE cells alone, whereas one signal (*m/z* 184) was found to be produced by omental tissue based on the spatial distribution (Fig. 1D; Supplementry Fig. S5). Signals were putatively annotated by searching for metabolites that matched the experimental monoisotopic mass in the Human Metabolome Database (Table 1; Supplementary Table S1; refs. 36, 37). It is worth noting that all assignments are designated as putative because of the nature of the parts per million error achieved from our MSI samples. It is a well-documented effect that the slight surface heterogeneity and added height from the glass slides contribute to the higher mass errors observed. Putative annotations for these signals suggested alterations in amino acid metabolism and the potential induction of catecholamine signaling in cocultures (Table 1). Valine and histidine are both essential amino acids, and epinephrine is a catecholamine neurotransmitter (38).

**Table 1:** Putative annotations and parts per million error

MSI signal (observed)	Putative adduct	Exact mass (calc)	Putative annotation	ppm error
*m/z* 118.0918	(M + H)^+^	118.0863 Da	Valine	46.6
*m/z* 140.0735	(M + Na)^+^	140.0682 Da	Valine	37.8
*m/z* 156.0470	(M + H)^+^	156.0768 Da	Histidine	−191
*m/z* 184.0775	(M + H)^+^	184.0968 Da	Epinephrine	−105

Abbreviations: calc, calculated; ppm, parts per million; [M+H]^+^, protonated molecule; [M+Na]^+^, sodiated adduct.

Signals (*m/z*) identified in the initial MSI screen and putative annotations based on the adduct and searching the expected monoisotopic mass in the Human Metabolome Database (ref. 38). The parts per million error was calculated based on the measured accurate mass and the calculated exact mass of putatively annotated metabolites in their observed adduct form.

### Valine annotation was confirmed using tandem mass spectrometry

Although we were able to identify spatially interesting signals and determine their relative abundance using MSI, we were unable to obtain high-quality fragmentation patterns directly from the MSI sample because of low overall signal intensity and ion suppression within the agarose culture. To obtain fragmentation patterns, metabolites were extracted from agarose cocultures using organic solvents, and the extracts were resuspended at 1 mg/mL (Supplementry Fig. S6A). Extracts were then analyzed alongside commercial standards of putatively identified metabolites using the dried droplet method for MALDI/tandem mass spectrometry (Supplementry Fig. S6A). The fragmentation pattern for *m/z* 118 in the tumorigenic FTE cell/omentum coculture matched the fragmentation pattern from L-valine (Supplementry Fig. S6B), thus confirming the putative annotation with level 2 confidence (39, 40).

### Amino acids quantified using LC/MS showed that BCAA catabolism is increased in tumorigenic FTE cell/omentum cocultures

**Figure 2:**
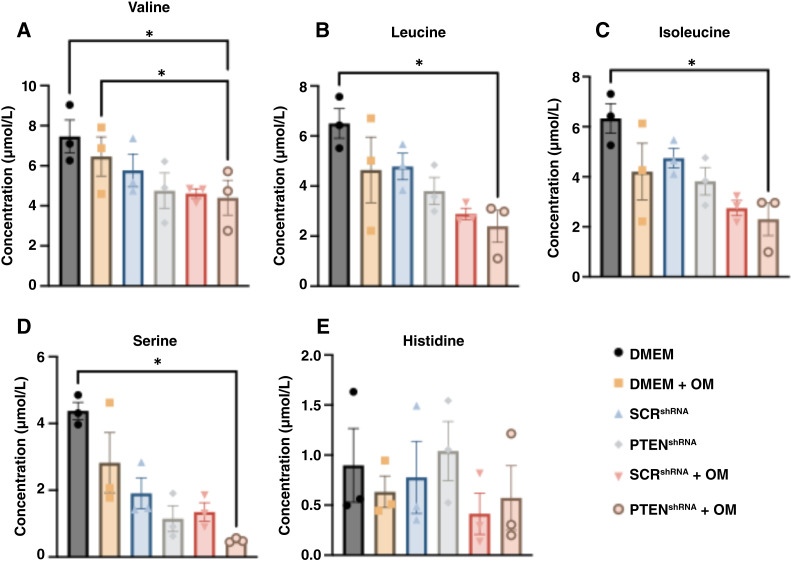
LC/MS data show increased consumption of BCAAs and serine and no change in histidine concentration under co-culture with omentum (OM). LC/MS results from the analysis of 3D agarose coculture extracts using the aTRAQ kit to determine concentrations of (**A**) valine, (**B**) leucine, (**C**) isoleucine, and (**D**) serine, and (**E**) histidine in extracts resuspended at 1 mg/mL. Significance was determined using one-way ANOVA with the Tukey *post hoc* test (*, *P* < 0.05).

To orthogonally validate putative annotations and further probe alterations in amino acid metabolism, amino acids in coculture extracts (*N* = 3) were quantified using an aTRAQ kit (Sciex) with LC/MS analysis (27, 28). Overall, we could detect and quantify 24 amino acids by retention time matching with internal standards. Thus, each amino acid was identified with level 1 confidence (Supplementary Table S1; ref. 39). Serine was significantly decreased (*P* < 0.05) in the tumorigenic FTE cell/omentum condition compared with the DMEM control (Fig. 2D). Interestingly, data on valine and other BCAAs, including leucine and isoleucine, revealed decreased levels in the tumorigenic FTE cell/omentum condition compared with media alone (Fig. 2A–C; ref. 41). The aTRAQ kit is capable of quantifying 44 different amino acids and amino acid derivatives; of these, 24 were detected and quantified in coculture extracts, and 20 were not detected (Supplementry Table S2; Supplementry Fig. S7).

### Analysis of the valine calibration curve using MSI shows evidence of ion suppression

Based on the aforementioned results, we tested the hypothesis that ion suppression caused the apparent “increase” in valine levels in tumorigenic FTE/omentum cocultures (Fig. 1B; Supplementry Fig. S3), whereas our quantitative LC/MS data showed a decrease (Fig. 2A). Ion suppression is a type of matrix effect in which certain metabolites are inhibited from being ionized and, consequently, remain undetected. This phenomenon can arise because of various factors, including the presence of highly abundant metabolites that compete for ionization (42). As a result, some metabolites may exhibit a linear response, with their signal intensity increasing as concentrations increase until reaching a threshold. Beyond that threshold, the signal intensity begins to decrease with further increases in concentration.

**Figure 3:**
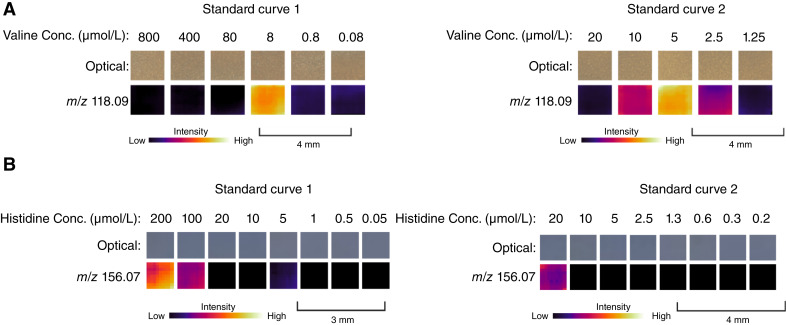
Ion suppression tests for select amino acids in cell culture media. **A,** Ion images showing signal intensity of *m/z* 118.09 with different concentrations (Conc.) of a valine analytic standard spiked into agarose. Standard curve 1 shows concentrations ranging from 800 to 0.08 μmol/L, whereas standard curve 2 shows concentrations ranging from 20 to 1.25 μmol/L. DMEM contains 800 μmol/L L-valine. **B,** Ion images showing signal *m/z* 156.07 with different concentrations (Conc.) of a histidine analytic standard spiked into agarose. Standard curve 1 shows concentrations ranging from 200 to 0.05 μmol/L, whereas standard curve 2 shows concentrations ranging from 20 to 0.2 μmol/L. DMEM contains 200 μmol/L L-histidine.

The media used for mammalian cell culture is supplemented with high concentrations of amino acids, including L-valine at a concentration of 800 μmol/L. We hypothesized that this high concentration of L-valine may induce ion suppression, and the consumption of L-valine in tumorigenic FTE/omentum cocultures may alleviate ion suppression, thereby leading to increased signal observed in MSI. We tested this hypothesis using standard curves with known concentrations of a valine analytic standard spiked into agarose. The first standard curve had concentrations of 800, 400, 80, 8, 0.8, and 0.08 μmol/L valine. MSI analysis revealed that no signal was observed from 800 to 80 μmol/L, and the highest signal intensity for *m/z* 118 was observed at the 8 μmol/L concentration. Below the 8 μmol/L concentration, the signal intensity dropped off, a result that is consistent with our hypothesis (Fig. 3A). For further confirmation, we analyzed a second standard curve with a 4× serial dilution of 20 μmol/L valine. Again, the signal intensity was low at the 20 μmol/L concentration, peaked at the 5 μmol/L concentration, and decreased at concentrations lower than 5 μmol/L. The intermediate concentrations, at 10 and 2.5 μmol/L, showed similar signal intensities for *m/z* 118, providing additional confidence that our hypothesis was confirmed (Fig. 3A). We noted that histidine also had a similar aTRAQ trend but was not significantly altered and subsequently does not suffer from ion suppression (Fig. 3B).

### Valine supplementation leads to increased proliferation and phosphorylation of mTOR in tumorigenic FTE cell cultures

**Figure 4:**
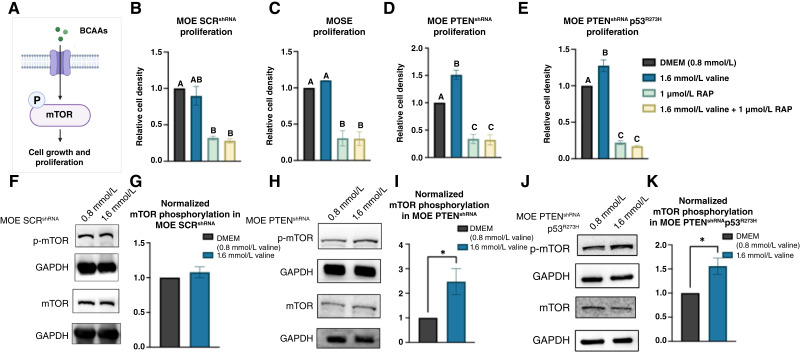
Valine supplementation increases the proliferation of tumorigenic FTE cells and activates mTOR signaling. **A,** Schematic of mTOR activation by BCAAs. **B–E,** Sulforhodamine B proliferation data from MOE SCR^shRNA^, MOSE, MOE PTEN^shRNA^, and MOE PTEN^shRNA^ p53^R273H^ cells in DMEM, 1.6 mmol/L valine DMEM, 1 μmol/L rapamycin (RAP), or combination. Significance was determined using one-way ANOVA with the Tukey’s *post hoc* test. Bars without the same letter (A–C) within **B–E** are significantly different from each other indicating *P* < 0.05. Representative Western blots and quantification of Western blotting (*N* = 3) showing increased phosphorylation of mTOR (p-mTOR) at 1.6 mmol/L valine in MOE SCR^shRNA^ (**F** and **G**), MOE PTEN^shRNA^ (**H** and **I**), and MOE PTEN^shRNA^ p53^R273H^ (**J** and **K**). Normalization was carried out first to the GAPDH loading control followed by total protein (mTOR). Significance was determined using a Student *t* test (*, *P* < 0.05). [**A,** Created in BioRender. Haughan, M. (2025) https://BioRender.com/nhtryoa.]

BCAAs are essential amino acids that mammalian cells cannot synthesize; hence, cancer cells likely acquire them through protein degradation or from the tumor microenvironment (TME). These amino acids play a fundamental role as building blocks for protein synthesis. They can be metabolized into branched-chain α-keto acids within the cytosol by branched-chain amino acid transaminase 1 (BCAT1) or within the mitochondria by branched-chain amino acid transaminase 2 (ref. 41). This process involves the conversion of α-ketoglutarate to glutamate. BCAAs may also serve as nitrogen sources for the biosynthesis of nucleotides and non-essential amino acids through the glutamate–glutamine axis (41). Additionally, they can be catabolized to produce acetyl-CoA and succinyl-CoA, which are utilized in the tricarboxylic acid cycle, thereby contributing to energy production (41). The BCAA leucine is a well-known mechanistic target of rapamycin (mTOR) agonist, and studies indicate that other BCAAs may also be capable of activating mTOR signaling (43, 44). mTOR functions, in part, by regulating the phosphorylation of p70 S6 kinase (ref. 45). This signaling pathway regulates proliferation and is often activated in tumors (46). We sought to characterize the impact of valine supplementation on proliferation and mTOR signaling in tumorigenic FTE cells as BCAAs are known to stimulate mTOR and proliferation in other cell types (Fig. 4A). We evaluated the proliferation of MOE SCR^shRNA^, MOSE, MOE PTEN^shRNA^, and MOE PTEN^shRNA^ p53^R273H^ cells in DMEM with valine concentrations of 800 μmol/L (0.8 mmol/L, standard DMEM concentration) and 1.6 mmol/L valine with or without 1 μmol/L rapamycin, a known mTOR inhibitor (Fig. 4B–E). We found that there was a significant increase in proliferation of tumorigenic MOE PTEN^shRNA^ cells (at day 5) and MOE PTEN^shRNA^ p53^R273H^ cells (day 3) when treated with 1.6 mmol/L valine-supplemented media compared with DMEM (Fig. 4D and E). No increase in proliferation was observed in the non-tumorigenic MOE SCR^shRNA^ or MOSE (Fig. 4B and C). These data indicate that 1.6 mmol/L valine supplementation increases the proliferation of tumorigenic FTE cells. Furthermore, we evaluated the phosphorylation of mTOR in 1.6 mmol/L valine-supplemented media after 24 hours by Western blotting. The results showed that the phosphorylation of mTOR increased with supplementation to 1.6 mmol/L valine (Fig. 4F–K), indicating that valine can stimulate the phosphorylation of mTOR in tumorigenic FTE cells, but not non-tumorigenic MOE SCR^shRNA^. Supplementation at 2.4 mmol/L did not show a dose-dependent effect (Supplementary Fig. S8).

## Discussion

In this study, we adapted our MSI protocol to investigate metabolic alterations at the interface of tumorigenic FTE cells and omental tissue to elucidate the chemical cues involved in secondary metastasis. Our analysis showed that several signals were elevated in tumorigenic FTE cell/omentum cocultures compared with the controls. Based on our initial screens, putative annotations suggested that amino acid metabolism was perturbed at this coculture interface. Notably, the signal at *m/z* 118 was confirmed to represent the BCAA valine, and subsequent quantitative LC/MS analysis revealed increased BCAA catabolism. We attributed the discrepancy between MSI and LC/MS results to ion suppression resulting from high concentrations of valine in the media. Furthermore, proliferation assays demonstrated that valine supplementation promotes the proliferation of tumorigenic FTE cells, and immunoblotting showed an increase in the phosphorylation of mTOR in two HGSOC cell lines.

Previous research has highlighted the role of amino acids in interactions between ovarian cancer cells and omental tissue (11, 17, 47–49). Aside from the increased consumption of BCAAs, another intriguing finding is the significant decrease in serine levels in tumorigenic FTE cocultures compared with media (Fig. 2). Serine plays a role in one-carbon metabolism, providing one-carbon units for DNA–histone methylation (50). Serine is also involved in glutathione biosynthesis, a metabolite previously reported to be increased in the ovarian cancer TME (50–52). Surprisingly, although histidine was identified as a putative MSI signal, it was found to be decreased in omental conditions with no difference in tumorigenic FTE compared with healthy FTE (Fig. 2). We did not observe increases in the levels of arginine or citrulline, which a previous study reported being secreted into the ovarian cancer TME (11, 17). The most notable discovery from our quantitative LC/MS data is the heightened consumption of valine and other BCAAs, which contradicted the initial MSI results.

The discrepancy between increased BCAA ionization in MALDI-MSI and decreased BCAA quantity in LC/MS was ascribed to a phenomenon known as ion suppression. Ion suppression is a well-known phenomenon that can reduce signal in both MALDI-MSI and LC/MS (42, 53). In mass spectrometry, a sample’s molecular complexity affects metabolites’ desorption and ionization (53). At elevated concentrations, certain metabolites hinder their own ionization, causing signal intensity to increase until reaching a threshold concentration, beyond which it decreases. Through valine standard curves, we determined that the increase in signal observed in MSI was due to the relief of ion suppression resulting from valine consumption in tumorigenic FTE/omentum cocultures. By extracting metabolites from omental cocultures and diluting them to 1 mg/mL, we reduced the sample complexity and valine concentration in LC/MS samples, thereby mitigating ion suppression. This discovery holds significant implications for future MSI investigations; when a metabolite of interest is known to have a high concentration in the media, researchers should consider analyzing a standard curve to assess the impact of ion suppression. Furthermore, this underscores the importance of orthogonally validating MSI results.

The catabolism of valine and other BCAAs was observed to be higher in tumorigenic FTE cell/omentum cocultures compared with the media. Notably, although the difference in BCAA levels between tumorigenic FTE and healthy FTE cocultures was not statistically significant in extracts by LC/MS, MSI data in a divided chamber format revealed a more pronounced effect in cells closely situated to omental tissue (Fig. 1D). This observation suggests that the increased BCAA catabolism is a local change related to proximity between cells and the tissues, likely driven by small molecule diffusion (chemical gradients). Additionally, this finding contextualizes why the effect was not significant between tumorigenic and nontumorigenic coculture extracts, because the averaging of the whole agarose plug, including all the FTE cells, in which distant cells from the omentum did not result in changes as seen in the MSI (Fig. 1D). Elevated BCAA catabolism in ovarian cancer is substantiated by previous studies demonstrating increased expression of the BCAA catabolic enzyme BCAT1 in HGSOC (54, 55). Knockdown of BCAT1 has been shown to repress growth, supporting its significance in cancer progression (54, 55). In a study by Zhang and Han (56), elevated levels of BCAT1 were found to stimulate proliferation and mTOR activity in breast cancer. These findings are consistent with our proliferation data, which showed that valine supplementation increased proliferation and the phosphorylation of mTOR in tumorigenic FTE cell cultures. Given our finding that leucine and isoleucine catabolism was also increased in co-culture, future studies exploring whether all these BCAAs increase proliferation and mTOR phosphorylation in tumorigenic FTE cells are warranted. When hyperactivated, mTOR signaling promotes cell proliferation and metabolism that aids in tumor progression; mTOR signaling is enhanced in various types of cancer, and inhibition of this pathway is a promising therapeutic target (57).

In further exploring the potential impacts of the observed increase in valine consumption, we employed both the MOE PTEN^shRNA^ and MOE PTEN^shRNA^ p53^R273H^ cell lines as we have previously demonstrated that genetic alterations beyond the knockdown of PTEN may be required to see a response to the metabolic alterations induced by co-culture (24). The use of a purely murine system allowed for the exclusion of cross-species interactions, but it constitutes a limitation as the current findings have yet to be validated in a human model. Thus, we intend to build on this work by exploring the interaction between human omental tissue and human cell lines, including in 3D, in future studies to assess the translational relevance of these results. Furthermore, although our results suggest that rapamycin decreases ovarian cancer cell proliferation, the effects of rapamycin and other mTOR inhibitors are still yet to be fully understood. Additionally future studies are needed to elucidate the specific cell types within the omentum responsible for the observed metabolic changes as the omental tissue contains a variety of immune cells, fibroblasts, and endothelial cells in addition to adipocytes.

### Conclusion

Our study sheds light on the metabolic dynamics implicated in the secondary metastasis of HGSOC to the omentum. Using MSI analysis, we uncovered alterations in amino acid metabolism and observed an increase in BCAA catabolism at the interface of ovarian cancer cells and omental tissue. This heightened BCAA catabolism may facilitate increased proliferation of ovarian cancer cells through mTOR phosphorylation at the omentum/cancer cell interface. Moving forward, our research will focus on investigating other MSI signals and metabolites identified in this study, as well as delving into the molecular mechanisms underlying the observed increase in BCAA catabolism at this interface. By elucidating the intricate interplay of chemical cues involved in secondary metastasis, our findings enhance our understanding of ovarian cancer pathogenesis and hold promise for the development of novel therapeutic strategies targeting the metastatic TME in HGSOC.

## Supplementary Material

Supplementary Figure 1 Figure S1. Omentum coculture drying photo.

Supplementary Figure 2 Figure S2. Live dead staining of omental tissue

Supplementary Figure 3 Figure S3. Omentum IMS replicates

Supplementary Figure 4 Figure S4. Other signals identified in the initial MSI screen

Supplementary Figure 5 Figure S5. Signals from Figure S4 that replicated when cells and omentum are physically separated in agarose.

Supplementary Figure 6 Figure S6. MALDI-MS/MS fragmentation data from tumorigenic FTE/omentum coculture extract reveals the signal at m/z 118 represents L-valine.

Supplementary Figure 7 Figure S7. LC-MS data from amino acids quantified using aTRAQ kit (Sciex).

Supplementary Figure 8 Figure S8. Proliferation and mTOR phosphorylation with supplementation to 2.4mM valine.

Supplementary Table 1 Supplementary Table 1. Putative annotations of other signals identified in the initial MSI screen.

Supplementary Table 2 Table S2. Ppm errors for amino acids quantified using aTRAQ kit
