# Supplementary material for: Branched-Chain Amino Acid Catabolism Promotes Ovarian Cancer Cell Proliferation via Phosphorylation of mTOR
Source: Cancer Res Commun. 2025 Apr 7;5(4):569–79. doi: 10.1158/2767-9764.CRC-24-0532 (PMC11973964; doi:10.1158/2767-9764.CRC-24-0532)

**Figure S1.** Omentum coculture drying photo. A) Bisected murine omentum from a mouse aged 6-8 weeks placed in the corner of the plug. Representative of the final MSI co-culture set-up selected. B-D) Different amounts of murine omentum placed in different locations in the agarose plug. All omental tissue did not dry flat but crystallized when desiccated using heat regardless of size.


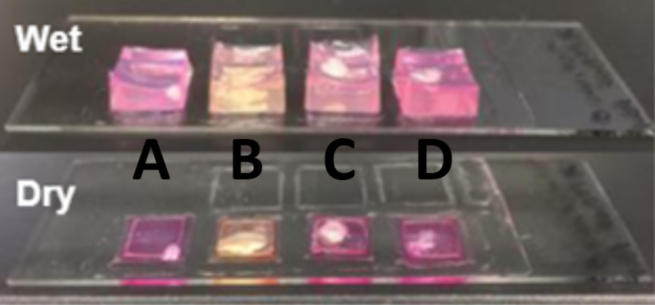

Supplement: Supplementary Figure 1 — Figure S1. Omentum coculture drying photo. [file crc-24-0532_supplementary_figure_1_suppsf1.docx]
