# Supplementary material for: Branched-Chain Amino Acid Catabolism Promotes Ovarian Cancer Cell Proliferation via Phosphorylation of mTOR
Source: Cancer Res Commun. 2025 Apr 7;5(4):569–79. doi: 10.1158/2767-9764.CRC-24-0532 (PMC11973964; doi:10.1158/2767-9764.CRC-24-0532)
Supplement: Supplementary Figure 2 — Figure S2. Live dead staining of omental tissue [file crc-24-0532_supplementary_figure_2_suppsf2.docx]

**Figure S2.** Live/Dead staining of omental tissue after 24 hours at 4°C and after 4 days at 37°C with Calcein AM (Live Green) and BOBO-3 Iodide (Dead Red). Scale bar 0.1mm.

**
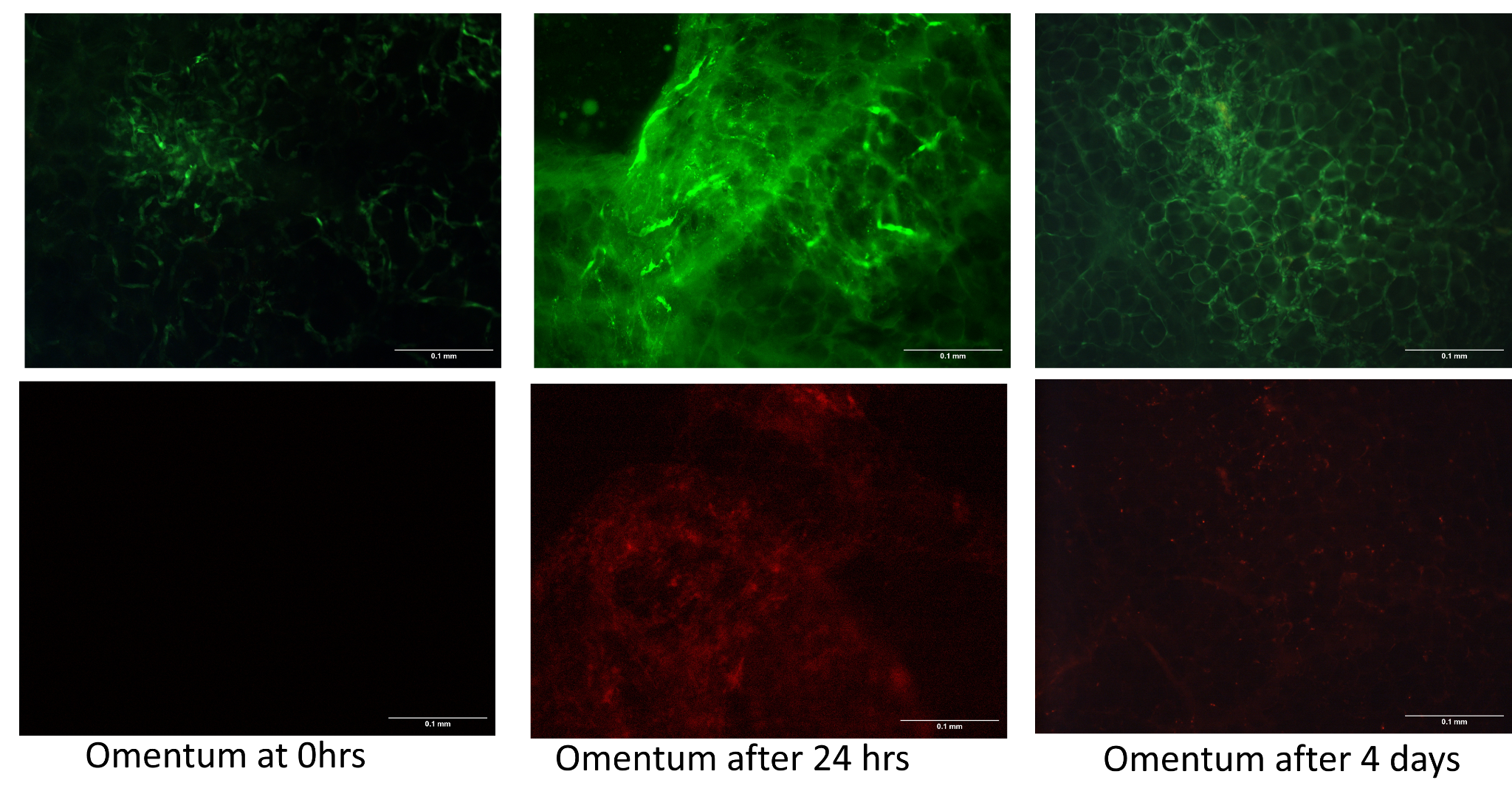
**
