# Supplementary figures and images for: Branched-Chain Amino Acid Catabolism Promotes Ovarian Cancer Cell Proliferation via Phosphorylation of mTOR
Source: Cancer Res Commun. 2025 Apr 7;5(4):569–79. doi: 10.1158/2767-9764.CRC-24-0532 (PMC11973964; doi:10.1158/2767-9764.CRC-24-0532)

**Figure S3.** Omentum IMS replicates (N=3).


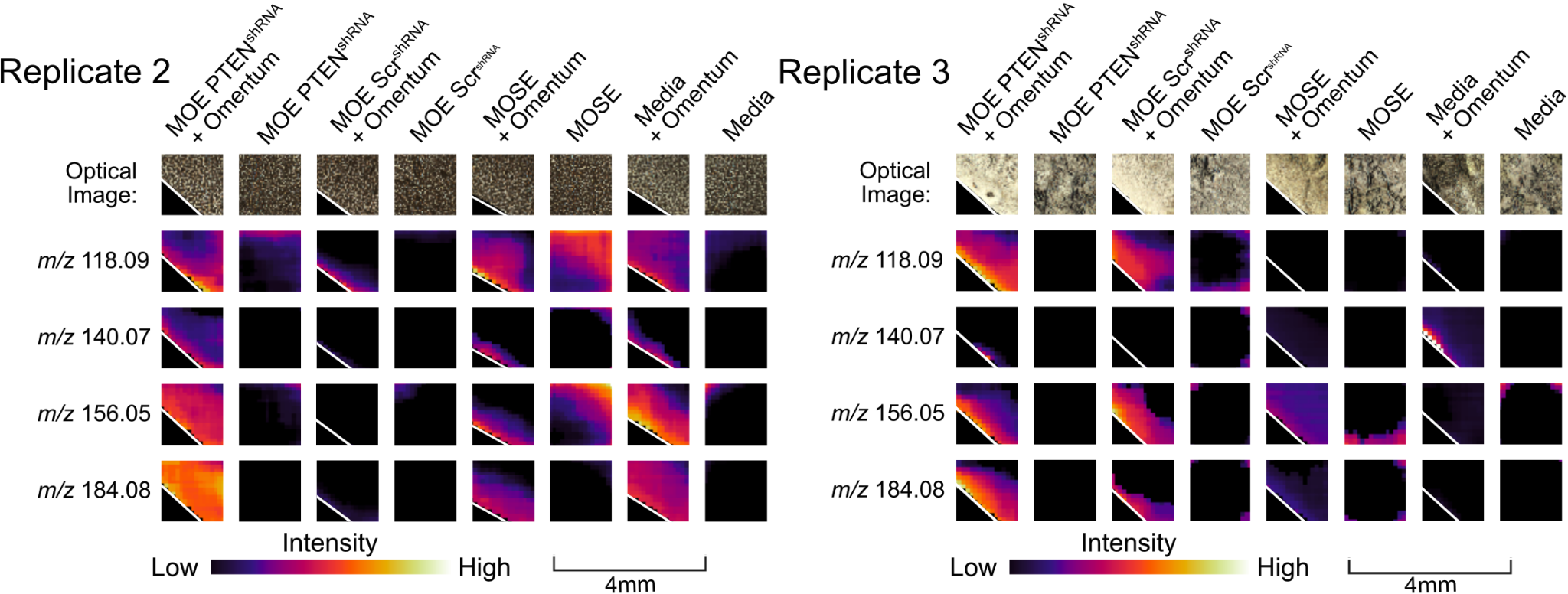

Supplement: Supplementary Figure 3 — Figure S3. Omentum IMS replicates [file crc-24-0532_supplementary_figure_3_suppsf3.docx]

**Figure S8.** Proliferation and mTOR phosphorylation with supplementation to 2.4mM valine.


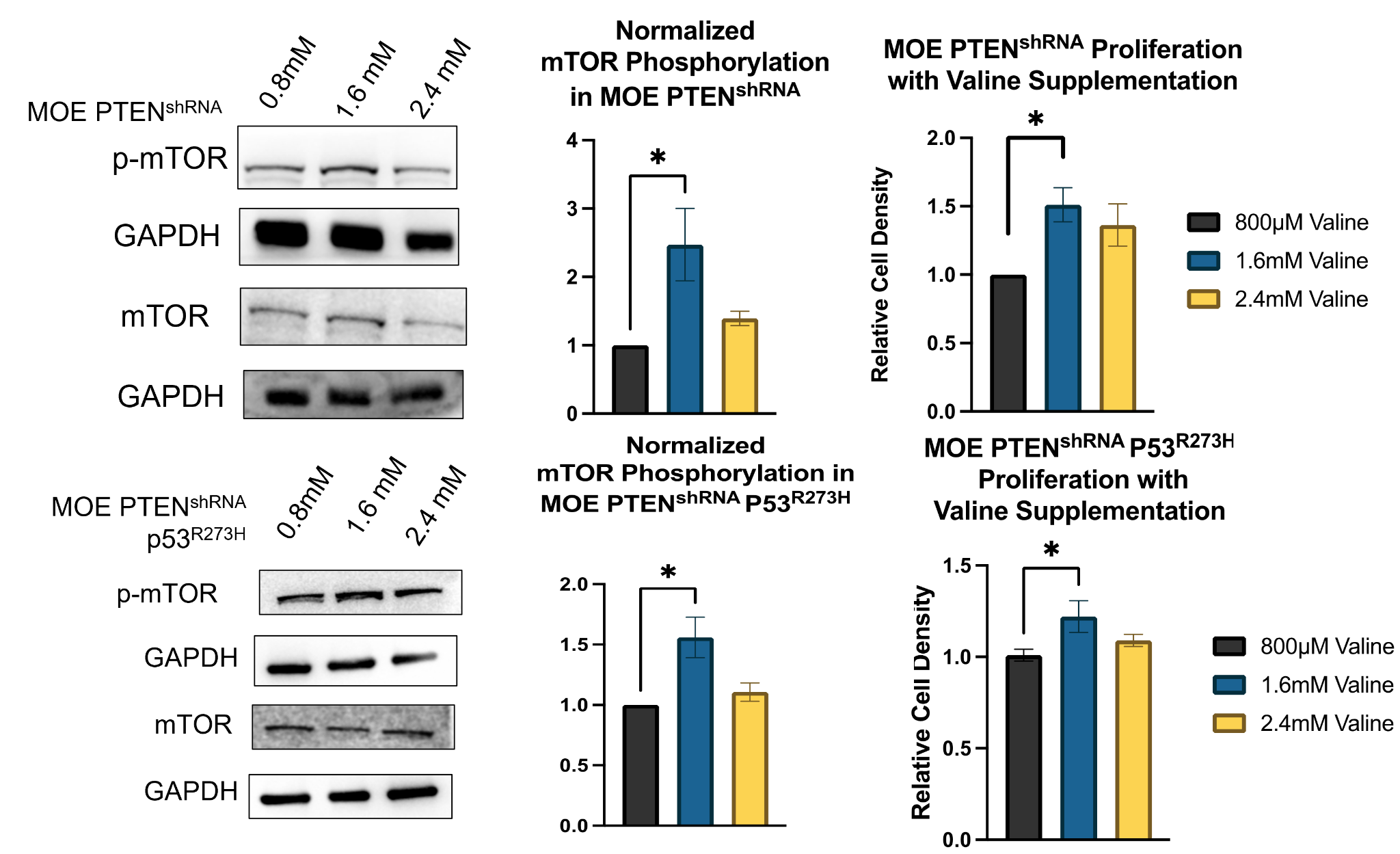

Supplement: Supplementary Figure 8 — Figure S8. Proliferation and mTOR phosphorylation with supplementation to 2.4mM valine. [file crc-24-0532_supplementary_figure_8_suppsf8.docx]
