# Supplementary material for: Branched-Chain Amino Acid Catabolism Promotes Ovarian Cancer Cell Proliferation via Phosphorylation of mTOR
Source: Cancer Res Commun. 2025 Apr 7;5(4):569–79. doi: 10.1158/2767-9764.CRC-24-0532 (PMC11973964; doi:10.1158/2767-9764.CRC-24-0532)
Supplement: Supplementary Figure 4 — Figure S4. Other signals identified in the initial MSI screen [file crc-24-0532_supplementary_figure_4_suppsf4.docx]

**Figure S4.** Other signals identified in the initial MSI screen (N=3). All signals are statistically significant compared to MOE PTEN^shRNA^ + omentum using Pearson's correlation analysis (p < 0.05).**
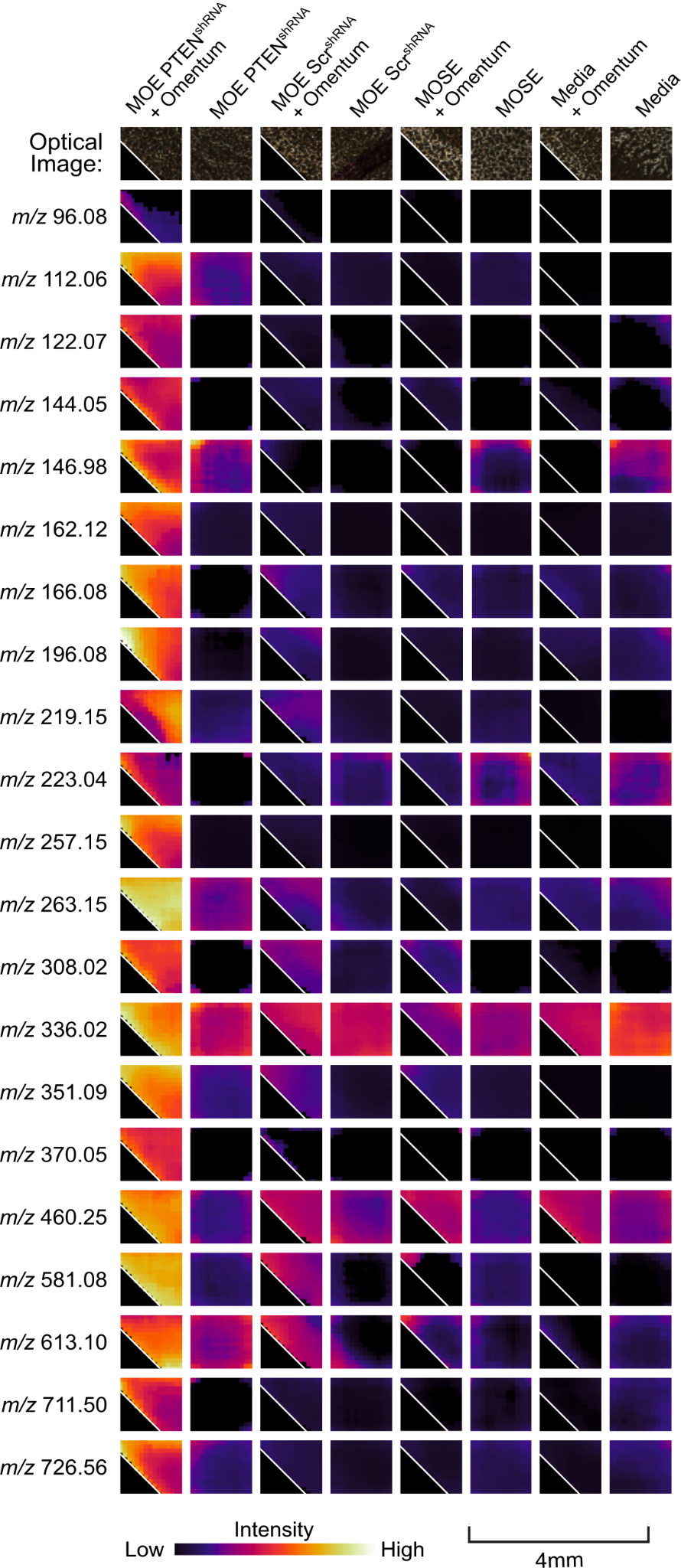
**
