# Supplementary material for: Branched-Chain Amino Acid Catabolism Promotes Ovarian Cancer Cell Proliferation via Phosphorylation of mTOR
Source: Cancer Res Commun. 2025 Apr 7;5(4):569–79. doi: 10.1158/2767-9764.CRC-24-0532 (PMC11973964; doi:10.1158/2767-9764.CRC-24-0532)
Supplement: Supplementary Figure 5 — Figure S5. Signals from Figure S4 that replicated when cells and omentum are physically separated in agarose. [file crc-24-0532_supplementary_figure_5_suppsf5.docx]

**Figure S5.** Signals from Figure S4 that replicated when cells and omentum are physically separated in agarose. Several signals originate from tumorigenic FTE cells, and several signals originate from omental tissue.

**
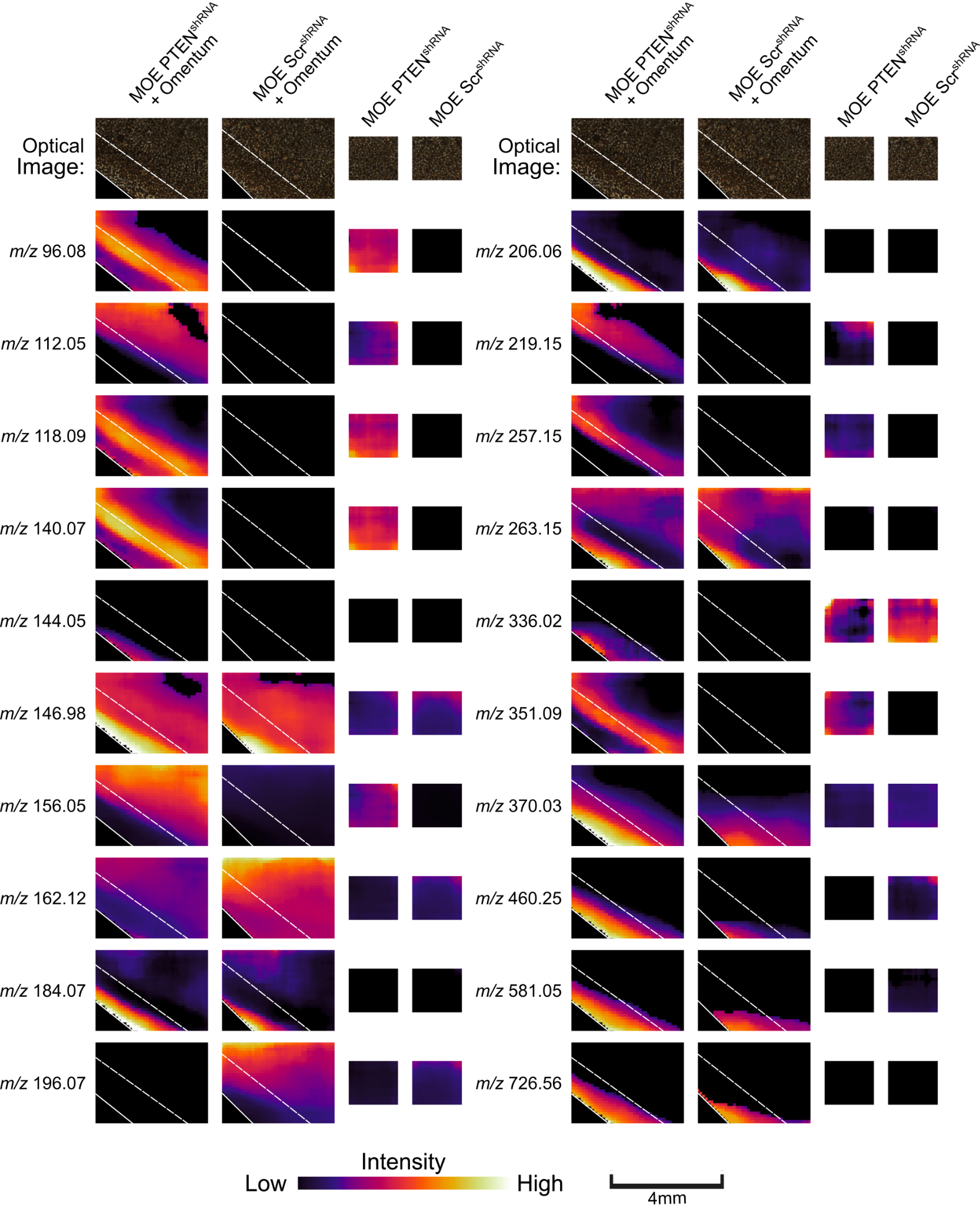
**
