# Supplementary material for: Branched-Chain Amino Acid Catabolism Promotes Ovarian Cancer Cell Proliferation via Phosphorylation of mTOR
Source: Cancer Res Commun. 2025 Apr 7;5(4):569–79. doi: 10.1158/2767-9764.CRC-24-0532 (PMC11973964; doi:10.1158/2767-9764.CRC-24-0532)

**Figure S6.** MALDI-MS/MS fragmentation data from tumorigenic FTE/omentum coculture extract reveals the signal at *m/z* 118 represents L-valine. **A)** Illustration of extraction procedure and MALDI-MS/MS analysis of tumorigenic FTE/omentum coculture extract **B)** Butterfly plot showing fragmentation patterns obtained from MALDI-MS/MS analysis of *m/z* 118 in the tumorigenic FTE/omentum coculture extract (top) and an L-valine analytical standard (bottom). The matching fragmentation pattern at 20 eV indicates that the IMS signal at *m/z* 118 represents L-valine.


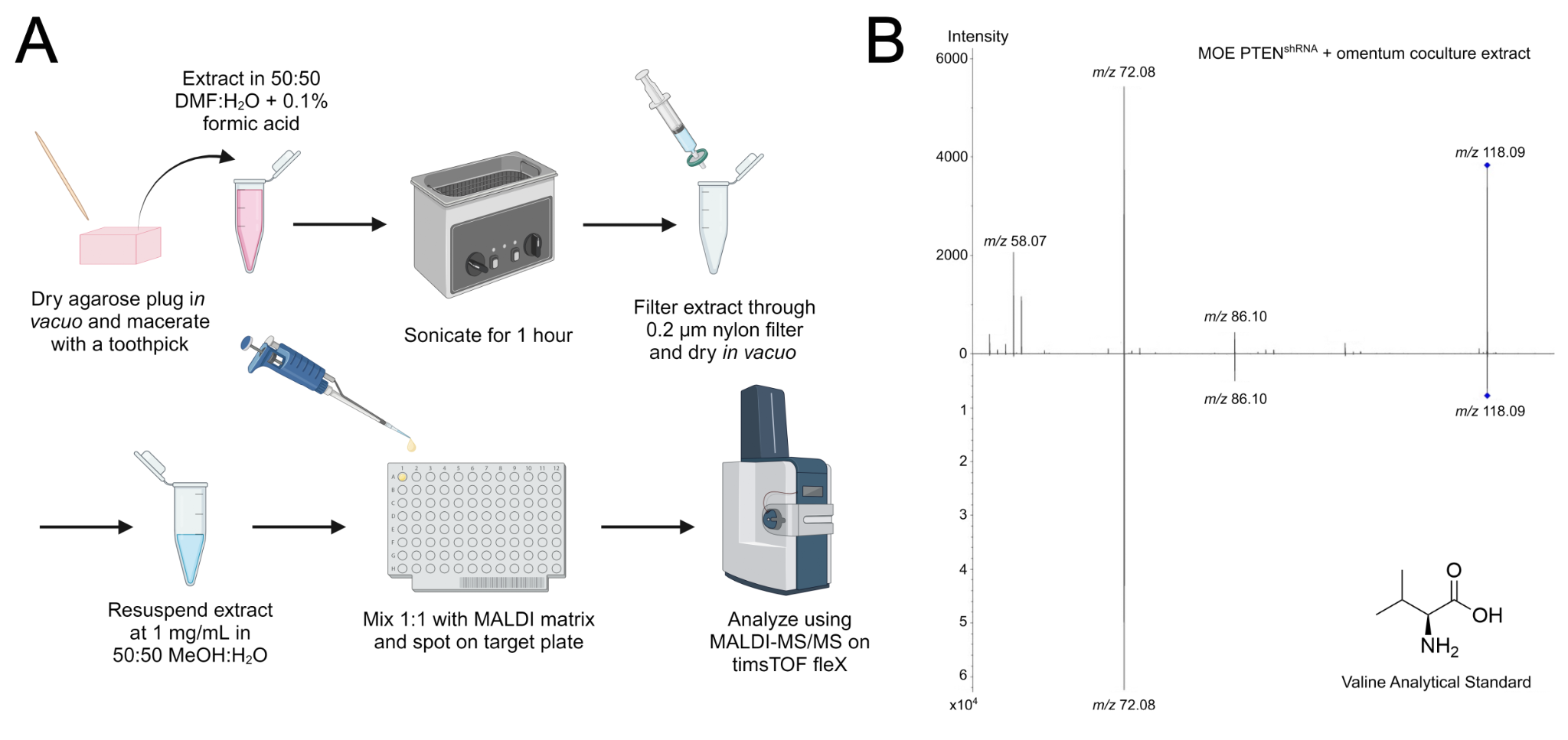

Supplement: Supplementary Figure 6 — Figure S6. MALDI-MS/MS fragmentation data from tumorigenic FTE/omentum coculture extract reveals the signal at m/z 118 represents L-valine. [file crc-24-0532_supplementary_figure_6_suppsf6.docx]
