# Supplementary material for: Branched-Chain Amino Acid Catabolism Promotes Ovarian Cancer Cell Proliferation via Phosphorylation of mTOR
Source: Cancer Res Commun. 2025 Apr 7;5(4):569–79. doi: 10.1158/2767-9764.CRC-24-0532 (PMC11973964; doi:10.1158/2767-9764.CRC-24-0532)

**Figure S7.** LC-MS data from amino acids quantified using aTRAQ kit (Sciex). Significance was determined using a one-way ANOVA with Tukey’s post hoc (* p<0.05).


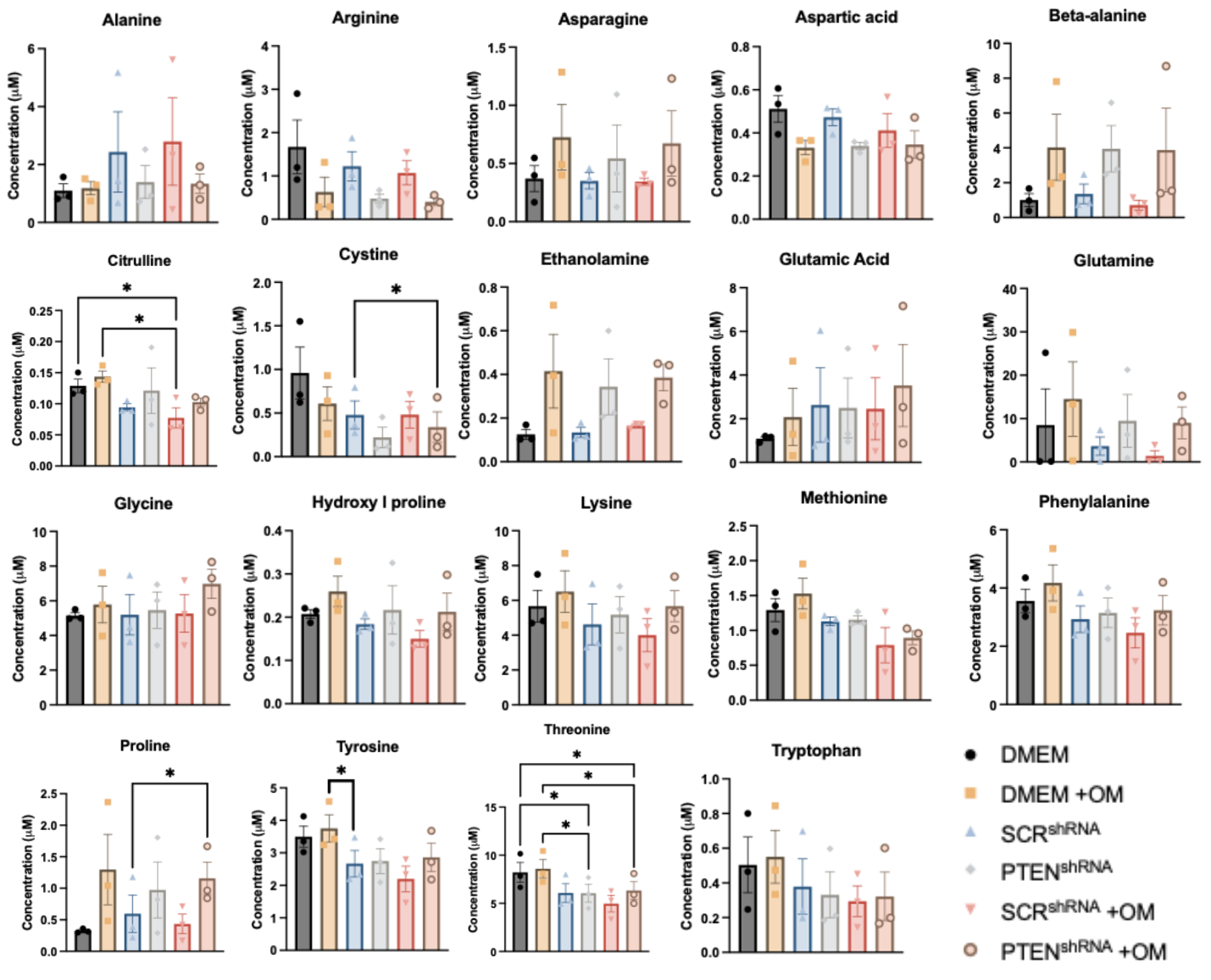

Supplement: Supplementary Figure 7 — Figure S7. LC-MS data from amino acids quantified using aTRAQ kit (Sciex). [file crc-24-0532_supplementary_figure_7_suppsf7.docx]
