# Supplementary material for: Branched-Chain Amino Acid Catabolism Promotes Ovarian Cancer Cell Proliferation via Phosphorylation of mTOR
Source: Cancer Res Commun. 2025 Apr 7;5(4):569–79. doi: 10.1158/2767-9764.CRC-24-0532 (PMC11973964; doi:10.1158/2767-9764.CRC-24-0532)
Supplement: Supplementary Table 1 — Putative annotations of other signals identified in the initial MSI screen. [file crc-24-0532_supplementary_table_1_suppst1.docx]

**Table S1.** Putative annotations of other signals identified in the initial MSI screen.

| **Signal** | **Adduct** | **Putative annotation** |
| --- | --- | --- |
| *m/z* 96.08 | [M+Na]^+^ | 2-Methyl-1-propylamine |
| *m/z* 112.06 | [M+H]^+^ | Histamine |
| *m/z* 122.07 | [M+H]^+^ | L-Cysteine |
| *m/z* 144.05 | [M+H]^+^ | Vinylacetylglycine |
| *m/z* 146.98 | [M+Na]^+^ | Phosphonoacetaldehyde |
| *m/z* 162.12 | [M+H]^+^ | L-Carnitine |
| *m/z* 166.08 | [M+H-H_2_O]^+^ | Epinephrine |
| *m/z* 196.08 | [M+H]^+^ | Dopaquinone |
| *m/z* 219.15 | [M+H]^+^ | N-acetylserotonin |
| *m/z* 223.04 | [M+H]^+^ | Cystathionine |
| *m/z* 257.15 | [M+K]^+^ | N-acetylserotonin |
| *m/z* 263.15 | [M+H]^+^ | Methylmalonylcarnitine |
| *m/z* 308.02 | [M+H]^+^ | Deoxycytidine monophosphate (dCMP) |
| *m/z* 336.02 | [M+H]^+^ | Dihydroneopterin phosphate |
| *m/z* 351.09 | [M+H]^+^ | Estrone sulfate |
| *m/z* 370.05 | [M+Na]^+^ | Adenosine monophosphate (AMP) |
| *m/z* 460.25 | [M+H]^+^ | N-Docosahexaenoyl Methionine |
| *m/z* 581.08 | [M+H]^+^ | Uridine diphosphate glucuronic acid |
| *m/z* 613.10 | [M+H]^+^ | Oxidized glutathione |
| *m/z* 711.50 | [M+H]^+^ | Phosphatidic acid (PA) |
| *m/z* 726.56 | [M+H]^+^ | Phosphatidylethanolamine (PE) or Phosphatidylcholine (PC) |
