# Supplementary material for: Branched-Chain Amino Acid Catabolism Promotes Ovarian Cancer Cell Proliferation via Phosphorylation of mTOR
Source: Cancer Res Commun. 2025 Apr 7;5(4):569–79. doi: 10.1158/2767-9764.CRC-24-0532 (PMC11973964; doi:10.1158/2767-9764.CRC-24-0532)
Supplement: Supplementary Table 2 — Table S2. Ppm errors for amino acids quantified using aTRAQ kit [file crc-24-0532_supplementary_table_2_suppst2.docx]

**Table S2.** Ppm errors for amino acids quantified using aTRAQ kit

| **Amino acid** | **Exact mass (calc)** | **Measured accurate mass** | **Error** |
| --- | --- | --- | --- |
| L-alanine | *m/z* 238.1641 | *m/z* 238.1620 | 8.8 ppm |
| L-arginine | *m/z* 323.2281 | *m/z* 323.2255 | 8.0 ppm |
| L-asparagine | *m/z* 281.1699 | *m/z* 281.1679 | 7.1 ppm |
| L-aspartic acid | *m/z* 282.1539 | *m/z* 282.1514 | 8.9 ppm |
| ꞵ-Alanine | *m/z* 238.1641 | *m/z* 238.1619 | 9.2 ppm |
| L-citrulline | *m/z* 324.2121 | *m/z* 324.2096 | 7.7 ppm |
| L-cystine | *m/z* 537.2495 | *m/z* 537.2441 | 10.1 ppm |
| Ethanolamine | *m/z* 210.1692 | *m/z* 210.1678 | 6.7 ppm |
| L-glutamic acid | *m/z* 296.1696 | *m/z* 296.1671 | 8.4 ppm |
| L-glutamine | *m/z* 295.1856 | *m/z* 295.1834 | 7.5 ppm |
| Glycine | *m/z* 224.1485 | *m/z* 224.1465 | 8.9 ppm |
| L-histidine | *m/z* 304.1859 | *m/z* 304.1833 | 8.5 ppm |
| Hydroxy-l-proline | *m/z* 280.1747 | *m/z* 280.1724 | 8.2 ppm |
| L-isoleucine | *m/z* 280.2111 | *m/z* 280.2090 | 7.5 ppm |
| L-leucine | *m/z* 280.2111 | *m/z* 280.2089 | 7.9 ppm |
| L-lysine | *m/z* 443.3311 | *m/z* 443.3273 | 8.6 ppm |
| L-methionine | *m/z* 298.1675 | *m/z* 298.1651 | 8.0 ppm |
| L-phenylalanine | *m/z* 314.1954 | *m/z* 314.1929 | 8.0 ppm |
| L-proline | *m/z* 264.1798 | *m/z* 264.1773 | 9.5 ppm |
| L-serine | *m/z* 254.1590 | *m/z* 254.1569 | 8.3 ppm |
| L-threonine | *m/z* 268.1747 | *m/z* 268.1726 | 7.8 ppm |
| L-tryptophan | *m/z* 353.2063 | *m/z* 353.2027 | 10.2 ppm |
| L-tyrosine | *m/z* 330.1903 | *m/z* 330.1875 | 8.5 ppm |
| L-valine | *m/z* 266.1954 | *m/z* 266.1933 | 7.9 ppm |
